# The Risk of Neutropenia and Leukopenia in Advanced Non-Small Cell Lung Cancer Patients Treated With Erlotinib: A Prisma-Compliant Systematic Review and Meta-Analysis

**DOI:** 10.1097/MD.0000000000001719

**Published:** 2015-10-09

**Authors:** Jian-Guo Zhou, Xu Tian, Long Cheng, Quan Zhou, Yuan Liu, Yu Zhang, Yu-ju Bai, Hu Ma

**Affiliations:** From the Department of Oncology, Affiliated Hospital of Zunyi Medical University (JGZ, LC,YZ,YJB, HM); Center for Translational Medicine, Zunyi Medical University, Zunyi (JGZ, HM); Graduate College (XT); School of Nursing, Tianjin University of Traditional Chinese Medicine, Tianjin (XT); Department of Science and Education, First People's Hospital of Changde City, Changde (QZ); Department of Pharmacology (YL); and Key Laboratory of Basic Pharmacology of Ministry of Education, Zunyi Medical University, Zunyi, China (YL).

## Abstract

Epidermal growth factor receptor-tyrosine kinase inhibitors (EGFR-TKIs) are a critical member of systemic therapy for advanced non-small-cell lung cancer (NSCLC). Erlotinib is the first-generation EGFR-TKIs, the National Comprehensive Cancer Network (NCCN) guidelines recommend it as a first-line agent in patients with sensitizing EGFR mutations. However, the safety of erlotinib plus chemotherapy (CT) or erlotinib alone for advanced NSCLC remains controversial. We carried out a systematic meta-analysis to determine the overall risk of neutropenia and leukopenia associated with erlotinib.

PubMed, EMBASE, CBM, CNKI, *WanFang* database, The Cochrane library, Web of Science, as well as abstracts presented at ASCO conferences and ClinicalTrials.gov were searched to identify relevant studies. RR with 95% CIs for neutropenia and leukopenia were all extracted. The random-effects model was used to calculate pooled RRs and 95% CIs. Power calculation was performed using macro embedded in SAS software after all syntheses were conducted.

We identified 12 eligible studies involving 3932 patients. Erlotinib plus CT or alone relative to CT is associated with significantly decreased risks of neutropenia and leukopenia in patients with advanced NSCLC (RR, 0.38; 95% CI, 0.21–0.71; *P* = 0.00; incidence: 9.9 vs. 35.2%) and (RR, 0.32; 95% CI, 0.11–0.93; *P* = 0.04; incidence: 3.5 vs. 11.6%), respectively. The subgroup analysis by erlotinb with or without CT showed that erlotinib combine with CT have no significance decrease the relative risks of neutropenia or leukopenia (RR, 0.98; 95% CI, 0.78–1.23; *P* = 0.87; incidence: 26.2 vs. 30.5%) and (RR, 0.81; 95% CI, 0.34–1.95; *P* = 0.64; incidence: 6.5 vs. 9.3%), respectively. However, erlotinib alone could decrease incidence of neutropenia (RR, 0.14; 95% CI, 0.07–0.27; *P* = 0.00; incidence: 3.7 vs. 40.8%) or leukopenia (RR, 0.07; 95% CI, 0.01–0.45; *P* = 0.01; incidence: 0.8 vs. 15.7%). The power analysis suggests that a power of 61.31% was determined to detect an RR of 0.38 for neutropenia, and 78.03% for an RR of 0.32 for leukopenia.

The present meta-analysis suggested that erlotinib could decrease the incidence of neutropenia and leukopenia in patients with advanced NSCLC undergoing erlotinib regardless of whether combined with CT or not. The subgroup analysis revealed that erlotinib combine with CT did not affect the incidence; however, erlotinib alone could significantly decrease the incidence of neutropenia and leukopenia compared with CT alone.

## INTRODUCTION

Lung cancer was the most frequently diagnosed cancer and the leading cause of cancer death among males in worldwide. Approximately 1.8 million new lung cancer cases were diagnosed in 2012.^1,2^ Current recommendations support epidermal growth factor receptor tyrosine kinase inhibitors (EGFR-TKIs) to treat the advanced non-small cell lung cancer (NSCLC) with EGFR-mutation. Erlotinib (Tarceva) is first generation of oral EGFR-TKIs.^3^ Before this work, we performed a meta-analysis to determine the efficacy of erlotinib combine with chemotherapy (CT) or alone in advanced NSCLC.^4^ Nevertheless, the toxicity of erlotinib in advanced NSCLC patients was not clear.

In addition, clinical trials have identified a series of adverse events (AEs) caused by EGFR-TKIs, in which acneiform eruption is the most frequently reported.^5–7^ In recent years, Shi et al and Qi et al^8,9^ accomplished meta-analyses to identify the risk of interstitial lung disease with EGFR-TKIs in advanced NSCLC, and another group finished a pooled analysis to determine the incidence and RRs of fatal AEs in cancer patients treated with EGFR-TKIs. However, hematologic toxicity is a common AE caused by CT agents. Life-threatening events (ie, severe infection, bleeding) might occur if decreased blood cells have not been managed timely.^10^ Especially, neutropenia and leukopenia were the critical prognostic factors in patients with cancers. Shitara et al's^11^ results suggest that neutropenia or leukopenia experienced during CT is associated with improved survival in patients with advanced cancers.

Therefore, a pooled analysis of the currently available studies restricted to patients who used erlotinib combine with chemotherapy or alone provides relevant information for neutropenia and leukopenia of patients with advanced NSCLC.

## METHODS

Ethical approval and patient written informed consent are not required as this is a systematic review and meta-analysis of previously published studies. This study was performed in accordance with the Preferred Reporting Items for Systematic Reviews and Meta-Analyses (PRISMA) statement.^12^ The protocol was published by Centre for Reviews and Dissemination PROSPERO (Registration No. CRD4201401335).

### Search Strategy

Eligible trials were identified through electronically searching the databases PubMed, China National Knowledge Infrastructure (CNKI), China Biomedical Literature database (CBM), EMBASE, Web of Science, and The Cochrane library using the following terms: (“non-small-cell lung carcinoma” OR “non-small cell lung cancer”) AND (“Erlotinib” OR “Tarceva”) (from inception to August 21, 2014, update in May 22, 2015). The search strategy for English language database was summarized in Appendix 1. American Society of Clinical Oncology conferences (ASCO) and ClinicalTrials.gov were also searched for relevant studies. Language or date restrictions were not imposed. We manually searched bibliographies of included trials and related reviews for additional references.^4^

### Selection Criteria

The following study selection criteria were applied. First, population: patients were diagnosed as having advanced NSCLC. No other restrictions were imposed; second, intervention: erlotinib plus chemotherapy or alone; third, comparison: chemotherapy alone; fourth, outcomes: hematologic toxicity will be evaluated; fifth, study design: RCTs.

### Data Extraction and Assessment for Risk of Bias

Two reviewers (J-GZ and LC) independently screened the titles and abstracts to exclude studies that failed to meet the inclusion criteria, and the full texts of the remaining were subsequently reviewed. Finally, data extraction was conducted using a premade data extraction form based on electronic database to collect information as follows: authors, the population studied, publication year, country, and the detailed information regarding PICOs. YZ performed the data extraction and entry, and YL was in charge of examining the data. Risk of bias of individual studies was assessed independently by J-GZ and XT with the Cochrane Collaboration's tool.^13^ We evaluated the following domains: random sequence generation, allocation concealment, blinding of participants and personnel, blinding of outcome assessment, incomplete outcome data, selective reporting, and other bias. Based on the information extracted from primary studies, each domain was rated as “high risk,” “unclear risk,” or “low risk.” Any disagreement between searchers concerning the eligibility of a trial was resolved by consulting a third reviewer (Y-JB).

### Statistical Analysis

We estimated the relative risk (RR) with 95% confidence interval (CI) for dichotomous outcomes. A random-effects model was used regardless of heterogeneity. Level of heterogeneity (level of variance) across studies was evaluated using *I*^*2*^ statistic. We considered heterogeneity substantial if a *I*^*2*^ ≥ 50%.^14^ In contrast, if the clinical characteristic and/or methodology across studies regardless *I*^*2*^ statistic was considered to be obviously different, and thus qualitative analysis was adopted.^15^ Subgroup and sensitivity analyses were conducted to determine the possible causes of heterogeneity and to further identify the influence of various exclusion criteria on the overall risk estimate.^16^ The presence of publication bias was evaluated by using the funnel plots, Begg and Egger tests.^17,18^ Power calculation was performed using the methodology described by Cafri et al^19,20^ after all syntheses were performed. Details on the macro and SAS code used were included in the online supplement.

We considered a *P* value of less than 0.05 to be statistically significant. Meta analyses were performed by using STATA version 12.0 (Stata Corp., College Station, TX) and risk of bias was appraised by Review Manager (RevMan) version 5.3.4 (The Nordic Cochrane Centre, Copenhagen, Denmark), the incidences of neutropenia and leukopenia were calculated by Meta-Analyst Version 3.13 (Tufts Medical Center, Boston, MA), and power analysis was performed by SAS version 9.21 (SAS Institute Inc, North Carolina, USA).

## RESULTS

### Literature Research and Characteristic of Studies

**FIGURE 1:**
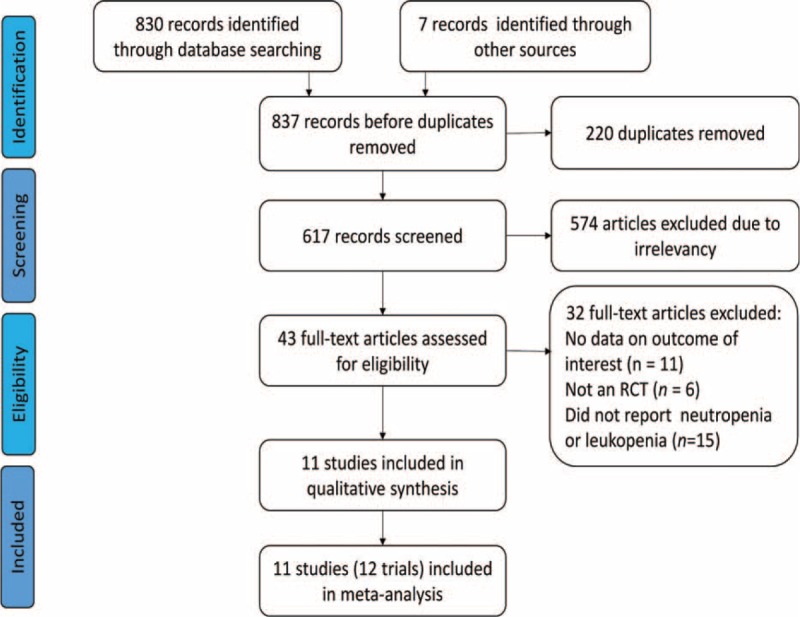
Flow diagram of the details of the study.

A total of 688 unfiled titles and abstracts were identified in the initial search, with 11 trails and 12 studies.^5–7,21–28^ A total of 3932 patients were enrolled of whom 1965 and 1967 patients were divided into erlotinib with or without CT and CT alone, respectively, meeting the inclusion criteria; thus, 2193 patients with NSCLC have appeared neutropenia, and 2800 patients have appeared leukopenia respectively, being included in the final analysis. The flow diagram of the literature searched and evaluated is presented in Figure 1.

All eligible studies were published between 2005 and 2015. In total, 12 studies provided outcomes; the trail finished by Lee et al^26^ was an RCT with 3-arm design comparing pemetrexed and erlotinib to either pemetrexed or erlotinib alone in patients with advanced NSCLC. Grade ≥3 neutropenia was available in 8 studies, 4 studies were erlotinib combine with CT, 4 studies were erlotinib-alone treatment with advanced NSCLC. Grade ≥3 leukopenia was appeared in 7 studies, erlotinib combine with CT and erlotinib alone were available in 4 and 3 studies, respectively. Ten studies reported AEs by grade according to the National Cancer Institute Common Terminology Criteria for Adverse Events (CTCAE) version 3.0, and Herbst et al's^24^ study has shown that the AEs were classified by CTCAE version 2.0; however, Gatzemeier et al's^23^ study did not report method to classify AEs. The main characteristics of the included studies are recorded in Table 1.

**TABLE 1:**
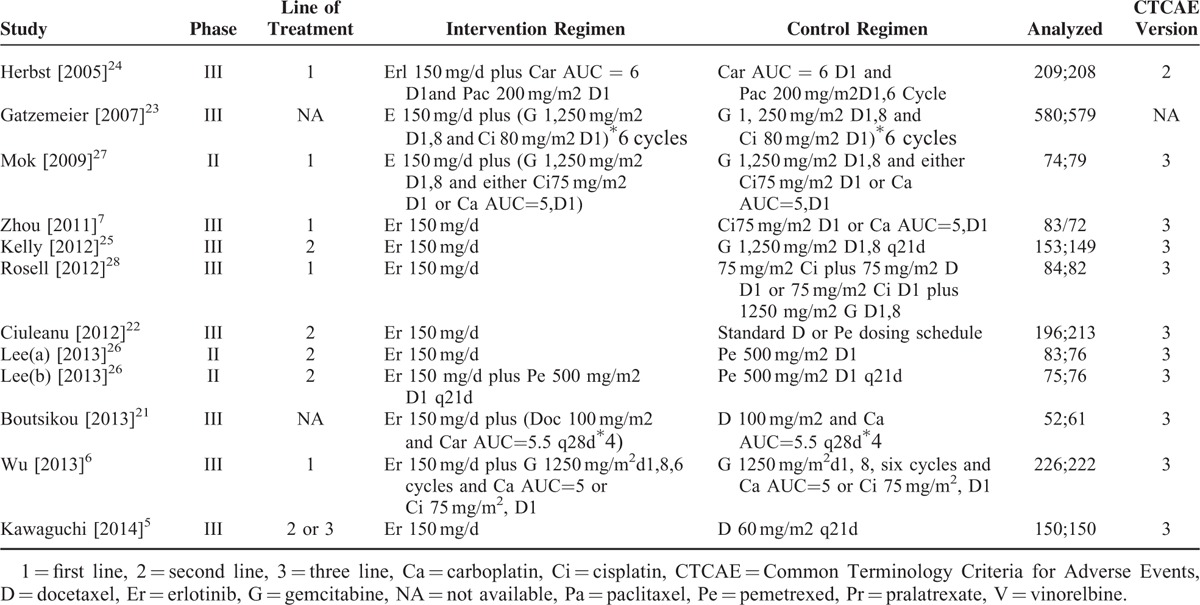
Main Characteristics of the Studies

### Assessing Risk of Bias

**FIGURE 2:**
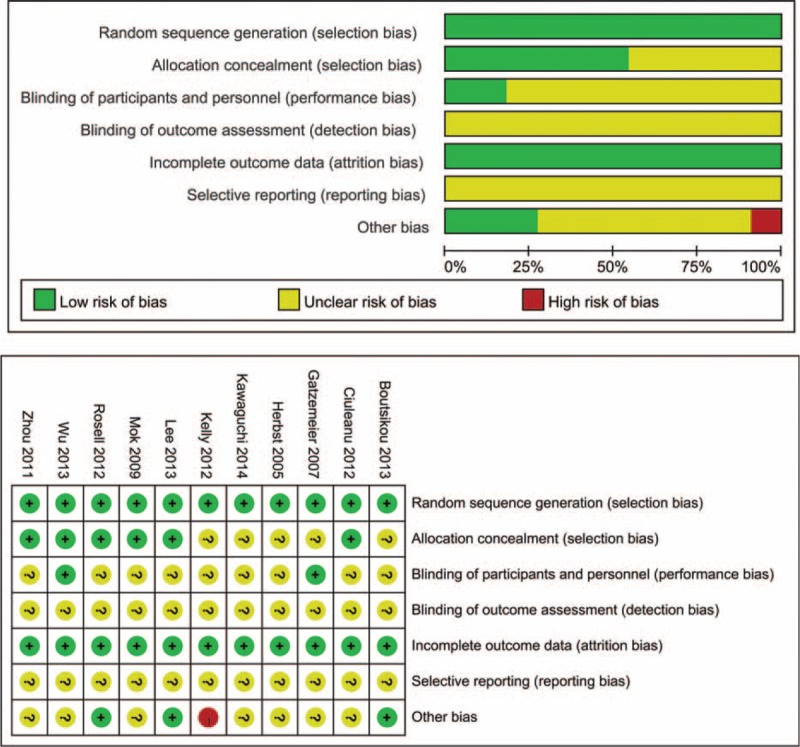
Appraisal of risk of bias of the included trials using the Cochrane risk-of-bias tool. Low risk = bias, if present, is unlikely to alter the results seriously, unclear risk = bias raises some doubt about the results, high risk = bias may alter the results seriously.

The detail of the risk-of-bias assessment is summarized in Figure 2. A total of 11 eligible trials were incorporated into the meta-analysis. All trials generated an adequate randomization sequence, and 6 trials^6,7,22,26–28^ presented appropriate allocation concealment. Only 2 trials^6,23^ performed appropriate blinding method to avoid performance bias. Detection bias resources did not exist in all the trials, but 1 trail^25^ has other potential; however, it was unlikely to affect the quality assessment. The overall methodological quality of the included trials was generally good and fair.

### Incidence and Relative Risk of Grade ≥3 Neutropenia Events

#### Incidence of Grade ≥3 Neutropenia

Eight RCTs reported the grade ≥3 neutropenia events, 4 RCTs were erlotinib alone, and 4 RCTs were erlotinib plus CT. In the erlotinib with or without the CT group, 219 patients experienced neutropenia compared with 420 patients in the CT group. The total incidence in erlotinib was 9.9% (95% CI, 4.2%–21.5%), and that of control was 35.2% (95% CI, 18.2%–57.0%). Subgroup analysis has shown that, in the combine group, erlotinib puls CT have lower incidences, which were 26.2% (95% CI, 14.1%–43.3%) and 30.5% (95% CI, 18.8%–45.3%) between 2 arms, respectively, in the erlotinib-alone group, erlotinib (RR, 3.7%; 95% CI, 1.8%–7.3%) compared with CT (RR, 40.8%; 95% CI, 7.1%–86.2%) has lower incidence of neutropenia.

#### Relative Risk of Grade ≥3 Neutropenia

**FIGURE 3:**
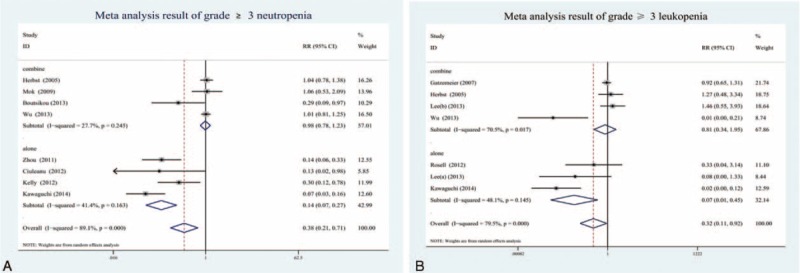
Meta-analysis result of the relative risk of neutropenia and leukopenia associated with erlotinib. Combined relative risk was calculated using the random-eVects model. A, The RR of grade ≥3 neutropenia. B, The RR of grade ≥3 leukopenia.

The heterogeneity test indicated that a random-effect model could be selected (*I*^*2*^ = 89.1%, *P* = 0.0). The pooled results showed that the erlotinib with or without CT group compared with CT group could decrease the relative risk of grade ≥3 neutropenia (RR, 0.38; 95% CI, 0.21–0.71); the result is presented in Figure 3A. No statistical significance was identified regarding the difference in neutropenia for subgroup by combine with CT (RR, 0.98; 95% CI, 0.78–1.23); however, the erlotinib alone could decrease ≥3 grade neutropenia compared with CT (RR, 0.14; 95% CI, 0.07–0.27).

### Incidence and Relative Risk of Grade ≥3 Leukopenia

#### Incidence of Grade ≥3 Leukopenia

Seven RCTs reported the grade ≥3 leukopenia events, analyzed 2800 patients, 3 RCTs were erlotinib alone, and 4 RCTs were erlotinib plus CT. In the erlotinib group, 74 patients experienced leukopenia compared with 221 patients in control group. The total incidence in erlotinib was 3.5% (95% CI, 1.6%–7.6%), and that of control was 11.6% (95% CI, 4.3%–27.7%). The lower incidence was observed in erlotinib puls CT group (0.8% vs. 15.7%). In the erlotinib alone group, incidence of neutropenia in erlotinib (RR, 6.5%; 95% CI, 3.2%–12.7%) compared with CT (RR, 9.3%; 95% CI, 4.9%–17.1%) did not have significant difference.

#### Relative Risk of Grade ≥3 Leukopenia

The heterogeneity test indicated that a random-effect model could be selected (*I*^*2*^ = 79.5%, *P* = 0.00). The pooled results showed that the erlotinib group compared with CT group could decrease the relative risk of grade ≥3 neutropenia (RR, 0.32; 95% CI, 0.11–0.93); the result is presented in Figure 3B. The subgroup analysis by erlotinib plus CT or alone suggested that erlotinib plus CT compared with CT have no statistical difference (RR, 0.81; 95% CI, 0.34–1.95), but the erlotinib alone could decrease ≥3 grade leukopenia in comparison with CT (RR, 0.07; 95% CI, 0.01–0.45).

### Sensitivity Analysis

**FIGURE 4:**
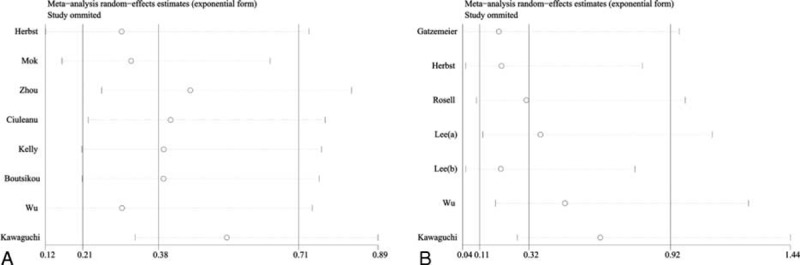
Sensitivity analysis result of relative risk of neutropenia and leukopenia associated with erlotinib. A, Grade ≥3 neutropenia. B, Grade ≥3 eukopenia.

Significant heterogeneity was observed among the included studies for leukopenia (*I*^*2*^ = 79.5%, *P* = 0.00) and neutropenia (*I*^*2*^ = 89.1%, *P* = 0.00). The subgroup analysis suggested that the heterogeneity of leukopenia between erlotinib combine with CT group and erlotinib alone group was not significance (*I*^*2*^ = 27.7%, *P* = 0.245 vs. *I*^*2*^ = 41.4%, *P* = 0.16); however, the heterogeneity of leukopenia between erlotinib combine with CT group and erlotinib alone group was *I*^*2*^ = 70.5% vs. *I*^*2*^ = 48.1%. As shown in Figure 4A, the study conducted by Wu et al^6^ showed results that were completely out of range of the others and probably contributed to the heterogeneity. After excluding this study, the results suggested that compared with CT, erlotinib plus CT could not increase the risk of leukopenia (RR, 1.00; 95% CI, 0.73–1.37). No evidence of high heterogeneity was observed among the remaining studies (*I*^*2*^ = 0.00%, *P* = 0.60).

### Power Analysis

Power calculations were performed post hoc after all of the studies had been collected using the methodology described by Cafri et al.^19^ We based on Zhou et al's^20^ study to analyze the power of relative risk of leukopenia and neutropenia. The power analysis suggests that power of 61.31% was determined to detect an RR of 0.38 for neutropenia, and 78.03% for an RR of 0.32 for leukopenia.

### Publication Bias

**FIGURE 5:**
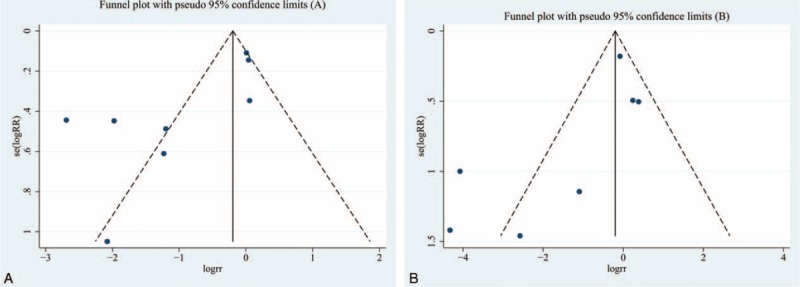
Funnel plots for the evaluation of publication bias. A, Grade ≥3 neutropenia. B, Grade ≥3 leukopenia. RR = relative risk, s.e. = standard error.

The publication bias of our meta-analysis was assessed using funnel plots, Begg, and Egger tests. As shown in Figure 5, no evidence of significant publication was found. There was no evidence of significant publication bias by inspection of the formal statistical tests [(1) neutropenia: Egger test, *P* = 0.266; Begg test, *P* = 0.020); (2) leukopenia: Egger test, *P* = 0.088; Begg test, *P* = 0.133)].

## DISCUSSION

EGFR-TKIs were one of the most important targeted agents, which could treat patients with EGFR mutation. Erlotinib was the first-generation ant-EGFR agent. Most of the meta-analyses focused on the effect of EGFR-TKIs^4,29–31^ have been published; some studies that determined the toxicity mostly focused on the fatal AEs such as treatment-related mortality,^32^ interstitial lung disease,^9^ skin rash,^33^ and gastrointestinal toxicities.^34^ To the best of our knowledge, this is the first meta-analysis to demonstrate a significantly decreased risk of ≥grade 3 neutropenia and leukopenia as a result of erlotinb-related treatment compared with CT. Many RCTs focused on the effect of erlotinib in advanced NSCLC,^5–7,21-28^ the effect including PFS, OS, objective response rate, and so on. However, the toxicity, especially neutropenia and leukopenia, did not report in all the completed clinical trials. Shitara et al suggested that neutropenia or leukopenia experienced during CT is associated with improved survival in patients with advanced cancers.^11^ The contribution of erlotinib to the risk of ≥grade 3 neutropenia and leukopenia was difficult to evaluate as individual RCT has not enough power to detect a significant difference compared with CT.

This meta-analysis enrolled 12 RCTs to overcome this limitation of underpowered, and demonstrated that the addition of erlotinib plus CT or alone to CT is associated with significantly decreased risks of neutropenia and leukopenia in patients with advanced NSCLC (RR, 0.38; 95% CI, 0.21–0.71; incidence: 9.9 vs. 35.2%) and (RR, 0.32; 95% CI, 0.11–0.93; incidence: 3.5 vs. 11.6%), respectively (Table 2). The subgroup analysis by erlotinb with or without CT showed that erlotinib combine with CT have no significance decrease the relative risks of neutropenia or leukopenia (RR, 0.98; 95% CI, 0.78–1.23; incidence: 26.2 vs. 30.5%) and (RR, 0.81; 95% CI, 0.34–1.95; incidence: 6.5 vs. 9.3%), respectively. However, erlotinib alone could decrease RRs of neutropenia (RR, 0.14; 95% CI, 0.07–0.27; incidence: 3.7 vs. 40.8%) or leukopenia (RR, 0.07; 95% CI, 0.01–0.45; incidence: 0.8 vs. 15.7%).

**TABLE 2:**
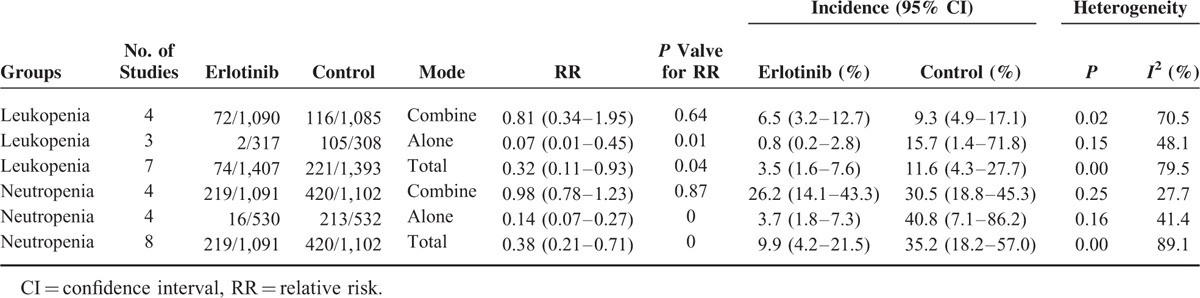
Incidence and Relative Risk of Grade ≥3 Hematologic Toxicity Events

Nonetheless, the relative risks of neutropenia and leukopenia were not serious, and Shitara et al suggested them as a preferential prognostic factor in patients with cancers undergoing chemotherapy.^11^ Unlike treatment-related death, skin rash, and gastrointestinal toxicities influence the effect that could be detected by every oncologist.

Our study has several strengths compared with the previously reported meta-analysis.^10^ To the best of our knowledge, this is the first systematic review and meta-analysis focused on the RRs of neutropenia and leukopenia in patients with advanced NSCLC undergoing erlotinib. In this study, power analysis for meta-analysis was used; the powers for the RR value of neutropenia and leukopenia were 61.31% vs. 78.03%, respectively, which discloses that there was sufficient evidence to clarify the results. Finally, there was little evidence of publication bias for both neutropenia and leukopenia.

We encountered several limitations during this meta-analysis, which need to be acknowledged. First, only a small number of eligible studies were included to assess the RRs of erlotinib alone versus CT, thus reducing the power of our study. Small sample size was the fatal shortcoming for all eligible studies and it might lead to an erroneous conclusion. Although no language restriction was imposed, some databases indexed in non-English and Chinese were not searched; it also contributed to selection bias. The overall methodological quality of the included trials was generally good and fair; however, most of the studies have defects of methodology. Inadequate methodology impaired the power of pooled results also.

Finally, the febrile neutropenia is a serious consequence of myelosuppressive CT that usually results in hospitalization and the need for intravenous antibiotics.^35^ Few studies focused on the incidence and RRs of febrile neutropenia in NSCLC with CT.^35–37^ We found 2 trials including febrile neutropenia,^24,28^ the heterogeneity test indicated that the RRs of febrile neutropenia have significant heterogeneity (*I*^*2*^ = 59.5%, *P* = 0.116), and in all studies have small sample size. Therefore, we give up the pooled analysis for febrile neutropenia.

In conclusion, the present meta-analysis suggested that erlotinib could decrease the RRs of neutropenia and leukopenia in patients with advanced NSCLC undergoing erlotinib regardless of combine with CT or alone. The subgroup analysis revealed that erlotinib combine with CT did not affect the RRs and incidence; however, erlotinib alone could significantly decrease the RRs of neutropenia and leukopenia compared with CT. However, our finding partly relies on studies, which have bias, and thus this conclusion should be interpreted cautiously. Therefore, high-quality and adequately powered RCTs for this subgroup of patients are warranted.
