# The Risk of Neutropenia and Leukopenia in Advanced Non-Small Cell Lung Cancer Patients Treated With Erlotinib: A Prisma-Compliant Systematic Review and Meta-Analysis: Erratum

**DOI:** 10.1097/01.md.0000475936.88100.3b

**Published:** 2015-12-28

In the article “The Risk of Neutropenia and Leukopenia in Advanced Non-Small Cell Lung Cancer Patients Treated With Erlotinib: A Prisma-Compliant Systematic Review and Meta-Analysis”,^1^ which appeared in Volume 94, Issue 40 of *Medicine*, author contributions to the article were not correctly presented. The article has since been corrected online.
